# Unterstützung für Familien mit einem psychisch erkrankten Elternteil: Welche Hilfen nehmen psychisch erkrankte Eltern in Anspruch und welche wünschen sie sich?

**DOI:** 10.1007/s00115-023-01584-3

**Published:** 2023-12-18

**Authors:** Stefanie Bienioschek, Daria Nolkemper, Jennifer Schroth, Joachim Behr, Martin Heinze, Ute Ziegenhain, Max Schmauss, Michael Kölch

**Affiliations:** 1 Klinik für Psychiatrie, Psychotherapie und Psychosomatik im Kindes- und Jugendalter, Universitätsklinikum Ruppin-Brandenburg, Fehrbelliner Str. 38, 16816 Neuruppin, Deutschland; 2 Medizinische Hochschule Brandenburg, Neuruppin, Deutschland; 3 Klinik für Kinder- und Jugendpsychiatrie und Psychotherapie, Universitätsklinikum Ulm, Ulm, Deutschland; 4 Klinik für Psychiatrie, Psychotherapie und Psychosomatik, Universitätsklinikum Ruppin-Brandenburg, Medizinische Hochschule Brandenburg Theodor Fontane, Neuruppin, Deutschland; 5 Klinik für Psychiatrie und Psychotherapie, Campus Mitte, Charité – Universitätsmedizin Berlin, Berlin, Deutschland; 6 Fakultät für Gesundheitswissenschaften, Gemeinsame Fakultät der Universität Potsdam, der Medizinischen Hochschule Brandenburg Theodor Fontane und der Brandenburgischen Technischen Universität Cottbus-Senftenberg, Potsdam, Neuruppin, Cottbus, Deutschland; 7 Zentrum für seelische Gesundheit, Immanuel Klinik Rüdersdorf, Universitätsklinikum der MHB, Rüdersdorf, Deutschland; 8 Klinik für Psychiatrie, Psychotherapie und Psychosomatik, Universität Augsburg, Augsburg, Deutschland; 9 Klinik für Psychiatrie, Neurologie, Psychosomatik und Psychotherapie im Kindes- und Jugendalter, Universitätsmedizin Rostock, Rostock, Deutschland

**Keywords:** Psychisch kranke Eltern, Hilfe und Unterstützungsangebote, Sozialgesetzbuch, Wunsch nach Unterstützung, Versorgungsqualität, Mentally ill parents, Support system, Social Security Code, Desires for support, Supply quality

## Abstract

**Hintergrund:**

In Deutschland besteht ein Hilfs- und Versorgungssystem für Familien, das sich aus verschiedenen Sektoren speist und vielfältige Angebote aufweist.

**Fragestellung:**

Erfassung der in Anspruch genommenen Unterstützungsangebote, Ermittlung kindbezogener Faktoren, die mit der Inanspruchnahme in Zusammenhang stehen, sowie der elterlichen Wünsche nach Hilfe.

**Material und Methode:**

Befragung von 160 (teil-)stationär in psychiatrischen Kliniken aufgenommenen Eltern in einem mündlichen Interview mittels standardisierter und teilstandardisierter Fragebögen.

**Ergebnisse:**

Die Ergebnisse zeigen, dass nichtprofessionelle Hilfe durch Familie und Freunde sowie ambulante Hilfen des fünften Sozialgesetzbuches (SGB V) am meisten in Anspruch genommen werden. Familien, in denen die Kinder als stärker auffällig eingeschätzt werden, nehmen mehr hochschwellige Hilfen in Anspruch als Familien mit Kindern, die weniger auffällig bewertet werden. Regionale Unterschiede gibt es insbesondere bei der Inanspruchnahme hochschwelliger Hilfen aus dem achten Sozialgesetzbuch (SGB VIII).

**Diskussion:**

Die Ergebnisse weisen darauf hin, dass Familien mit einem psychisch erkrankten Elternteil Hilfen erhalten und intensive Hilfen auch gerade bei stärker belasteten Familien ankommen. Jedoch gibt es regionale Unterschiede bei der Inanspruchnahme von und dem Wunsch nach Unterstützung.

## Abstract (en)

**Background:**

In Germany different offers of social support are available for families that are provided by different sectors, e.g., the youth welfare and the healthcare systems.

**Objective:**

Documentation of the utilized help, child-related factors that are associated with the utilization and the parental desires for support.

**Material and methods:**

Survey of 160 parents undergoing (partial) inpatient treatment in psychiatric hospitals via an oral interview using standardized and semi-standardized instruments.

**Results:**

The results show that nonprofessional help by family and friends as well as support offers provided by the healthcare system are used most frequently. Families that perceived their children as more burdened receive more help than families with children judged as being less burdened. There are regional differences especially in the utilization of high-threshold help by the healthcare system.

**Discussion:**

Support offers seem to reach families with mental illnesses, especially those that are particularly burdened; however, there are regional differences regarding the utilization of support as well as the wishes for specific support offers.

Psychisch kranke Eltern haben einen erhöhten Unterstützungsbedarf. Untersuchungen zeigen jedoch, dass die Hilfen nicht immer bei den Betroffenen ankommen. Die vorliegende Untersuchung erfasst, welche Unterstützungsangebote von Eltern mit einer psychischen Erkrankung in Anspruch genommen werden, welche Faktoren damit in Zusammenhang stehen und welche Hilfen von den Eltern gewünscht werden.

## Hintergrund

Kinder von Eltern mit einer psychischen Erkrankung haben ein erhöhtes Risiko, selbst eine psychische Erkrankung zu entwickeln [28]. Neben (epi-)genetischen Dispositionen sind es vor allem die mit der elterlichen Erkrankung einhergehenden psychosozialen Belastungen, die das Risiko für die Kinder erhöhen [15]. So zeigen Studien, dass Kinder von Eltern mit einer psychischen Erkrankung vermehrt Einschränkungen in der sozialen Teilhabe aufweisen [8]. Umgekehrt kann die soziale Teilhabe ein Resilienzfaktor für Kinder darstellen: Soziale Teilhabe dient als Indikator für Wohlbefinden und psychische Gesundheit [21, 22]. Kinder werden von ihren psychisch kranken Eltern doppelt so häufig als in ihrem Verhalten auffällig bzw. im Grenzbereich zur Auffälligkeit wahrgenommen als Kinder von gesunden Eltern [27]. Wirksamkeitsstudien zu zielgruppenspezifischen Interventionsprogrammen zeigen, dass psychisch erkrankte Eltern die (gesundheitsbezogene) Lebensqualität ihrer Kinder geringer einschätzen als gesunde Eltern, sich diese aber z. B. durch familienzentrierte Interventionen signifikant verbessern kann ebenso wie die soziale Unterstützung der Kinder [30].

In Deutschland besteht ein komplexes Unterstützungs- und Versorgungssystem. Von sehr niederschwelligen Bildungs- und Betreuungsangeboten wie Krabbelgruppen oder Jugendtreffs über therapeutische Angebote, Schul- und Familienhilfen bis hin zur stationären Aufnahme von Kindern in Wohngruppen oder medizinische Einrichtungen ist das Hilfesystem in Deutschland breit aufgestellt und wird aus unterschiedlichen Sektoren gespeist. Dabei sind verschiedene Sozialgesetzbücher für unterschiedliche Leistungen zuständig. Das 5. Sozialgesetzbuch (SGB V) beinhaltet vorrangig Leistungen für Krankenbehandlung, während das 8. Sozialgesetzbuch (SGB VIII) für Hilfen für Familien gedacht ist. Im 12. Sozialgesetzbuch (SGB XII) ist vorrangig die allgemeine Sozialhilfe abgedeckt. Trotz des breit aufgestellten Hilfesystems gibt es Hinweise darauf, dass Eltern diese nicht so umfänglich in Anspruch nehmen, dass es ihre Belastung reduziert [10, 26]. Empirische Studien zeigen, dass dies nicht an der fehlenden Wahrnehmung der Auffälligkeiten und eines Unterstützungs- oder gar eines Behandlungsbedarfs der Kinder durch die Eltern liegt. So weicht der Anteil der Eltern, die ihr Kind als behandlungsbedürftig einschätzt, erheblich von dem Anteil ab, der tatsächlich eine Behandlung in Anspruch nimmt [10]. Es scheint demnach eine Diskrepanz zwischen dem Bedarf an Unterstützung und Behandlung und der tatsächlichen Inanspruchnahme dieser zu geben [11]. Auch aus Sicht der betroffenen Eltern werden die in Anspruch genommenen Hilfen als nicht ausreichend wahrgenommen [29]. Verschiedene Studien zeigen, dass dies teilweise in der Unkenntnis spezifischer Angebote liegt [6, 29]. Es gebe auf Seiten der Familie aber auch verschiedene Ängste, wie bspw. vor einer Trennung vom Kind oder gar eines Sorgerechtsentzugs [6]. Auch in der Erhebung von Kölch und Schmid geben über die Hälfte der Befragten an, Ressentiments gegenüber dem Jugendamt zu haben [11]. Hefti et al. sehen die Problematik dabei eher in der Angebotsstruktur. Die Autorinnen nennen unzureichende interinstitutionelle Kommunikation, verschiedene Verantwortungsbereiche und mangelndes Wissen um die Bedürfnisse der betroffenen Familien als denkbare Gründe [6].

Zudem sind „gravierende ortsbezogene Differenzen bei der Gewährungspraxis bzw. Inanspruchnahme“ [17, S. 18] von SGB-VIII-Hilfen bekannt, die nicht allein durch einen erhöhten Bedarf in verschiedenen Regionen erklärt werden können [20]. So variiert bspw. die Zahl der Hilfen für Erziehung zwischen 72 und 924 Hilfen pro 10.000 unter 21-Jährige pro Jahr [ebd.]. Auch im Bereich des Gesundheitswesens zeigen sich deutliche Unterschiede in der Versorgungslage. Daten der Kassenärztlichen Bundesvereinigung zeigen, dass der Wohnort die Inanspruchnahme einer psychotherapeutischen Behandlung beeinflusst [9]. Für die spezifische Zielgruppe der psychisch kranken Eltern gibt es kaum Untersuchungen. In der einzigen aktuellen Erhebung zeigt sich, dass belastete Familien aus städtischen Regionen eher Hilfsangebote in Anspruch nehmen [18, 25, 27]. Es fehlen daher insbesondere für die Zielgruppe der schwer erkrankten Eltern systematische Untersuchungen zu den tatsächlichen Bedarfen und erhaltenen Hilfen, die in einer Versorgungsregion die Lücken in der Versorgung abbilden und indizierte Veränderungen in der Hilfestruktur identifizieren.

Vor dem Hintergrund des elterlichen Bedarfs an Unterstützung und dessen Bedeutung für eine gesunde kindliche Entwicklung soll in der vorliegenden Studie das Inanspruchnahmeverhalten von Hilfen von psychisch erkrankten Elternteilen, die sich in stationärer oder teilstationärer psychiatrischer Behandlung befinden, untersucht werden. Anhand dieser Bestandsaufnahme können Unterschiede in den regionalen Versorgungsstrukturen herausgearbeitet werden. Zusätzlich soll die Bewertung der erhaltenen Hilfen erfasst werden und welche Hilfsangebote sich die Befragten noch wünschen würden.

## Methodik

### Datenerhebung und Stichprobe

Die Stichprobe der psychisch kranken Eltern wurde in drei psychiatrischen Kliniken mit Versorgungsauftrag rekrutiert: In der Großstadt Augsburg (Bayern) und in der Kreisstadt Neuruppin (Brandenburg) sowie der Gemeinde Rüdersdorf (Brandenburg). Die Studienorte wurden aufgrund der erwarteten Unterschiede in Bezug auf die Verfügbarkeit von Unterstützungsmaßnahmen in städtischen und ländlichen Gebieten ausgewählt. Die beiden brandenburgischen Studienorte Neuruppin und Rüdersdorf wurden aufgrund vergleichbarer ländlicher Versorgungsstrukturen unter dem Studienort „Brandenburg“ zusammengefasst.

Die Rekrutierung erfolgte von 2016 bis 2018. Für die Erhebung lagen positive Voten der Ethikkommissionen der Kliniken vor. Es wurden alle Patienten und Patientinnen eingeschlossen, die Kinder unter 18 Jahren hatten und sich seit mindestens 3 Tagen in stationärer oder teilstationärer Behandlung befanden. Patienten und Patientinnen mit akuten psychiatrischen Störungen, die eine Teilnahme behinderten, oder mit eingeschränkter Einwilligungsfähigkeit wurden ausgeschlossen. Eltern mit mehreren Kindern wurden zu dem aus ihrer Sicht am stärksten belasteten Kind befragt.

Das mündliche Interview bestand aus zwei teilstrukturierten Fragebögen (der Fragebogen zu den Hilfsangeboten und zur Einschränkung der sozialen Teilhabe) und aus drei vollstrukturierten Fragebögen (der BSI, der ILK, der SDQ) sowie einem Teil zu soziodemografischen Daten. Das Interview fand in den Räumen der jeweiligen Klinik statt und dauerte 45–60 min.

### Erhebungsinstrumente

#### Hilfsangebote

Als Erhebungsinstrument wurde ein selbst entwickelter Fragebogen^1^ zur Erfassung der konkreten Form, Häufigkeit und Dauer verschiedener Hilfen, die Eltern für ihre Kinder in Anspruch genommen haben, eingesetzt. Der Fragebogen erfasst 4 Fragen zu verschiedenen Hilfen. Es wird nach 16 Hilfsangeboten konkret gefragt. Die in Anspruch genommenen Hilfen werden in dem semistandardisierten Fragebogen mit ja = 1 markiert und nach dem genauen Angebot dieser Kategorie gefragt. Nicht in Anspruch genommene Hilfen werden auf dem Fragebogen mit nein = 0 notiert (Beispielitem: Welche Hilfen und Unterstützungsangebote haben Sie mit oder für ihr Kind bisher [d. h. seit der ersten Behandlung aufgrund ihrer psychischen Erkrankung] in Anspruch genommen? Damit könnte zum Beispiel Folgendes gemeint sein: Beratung bei einer Erziehungs- und Familienberatungsstelle oder bei einer speziellen Jugendberatungsstelle? Beratung bei einer psychologischen Beratungsstelle? …).

Die professionellen Hilfen werden nach den entsprechenden deutschen Sozialgesetzbüchern klassifiziert und anhand der Schwere des Zugangs zur Hilfe, z. B. mit oder ohne Beantragung oder Überweisung, kategorisiert (sehr niederschwellige bis hochschwellige Hilfen). Beispiele für sehr niederschwellige Hilfen aus dem SGB VIII sind Krabbelgruppen, Kinderturnen oder Jugendzentren. Als niederschwellige Hilfen werden Beratungsstellen eingestuft. Beispiele für mittelschwellige Hilfen sind Schulbegleitung oder Maßnahmen der ambulanten Jugendhilfe wie eine Familienhilfe. Als hochschwellige Hilfen gilt u. A. die stationäre Jugendhilfe. Aus dem SGB V werden stationäre Therapien und Tageskliniken den hochschwelligen Hilfen zugeordnet.

In dem Fragebogen wird in Form einer offenen Frage ebenfalls danach gefragt, welche Unterstützung sich die Befragten gewünscht hätten (Item: Was hat Ihnen/Ihrem Kind gefehlt, welche Unterstützung hätten Sie sich für Ihr Kind/sich selbst gewünscht?).

#### Kindbezogene Faktoren

Die deutsche Version des Strength and Difficulties Questionnaire (SDQ-Deu; [5]), erfasst *individuelle Probleme und Stärken von Kindern* zwischen 6 und 15 Jahren anhand einer 3‑stufigen Likert-Skala (0 = nicht zutreffend, 1 = teilweise zutreffend, 2 = eindeutig zutreffend; [31, 32]). Um den Gesamtproblemwert anzugeben, werden 4 Skalen mit je 5 Items, die sich auf Probleme beziehen, aufsummiert.

Der Elternteil des Inventars zur Erfassung der *Lebensqualität* bei Kindern und Jugendlichen (ILK; [19]) ermöglicht u. A. die Berechnung eines Gesamtscores für die Lebensqualität (LQ 0–28). Die Antworten werden anhand einer 5‑stufigen Likert-Skala (1 = sehr gut, 2 = eher gut, 3 = teils/teils, 4 = eher schlecht, 5 = sehr gut) erfasst.

Ein selbst entwickelter Fragebogen [12] erfasst das Ausmaß der Beeinträchtigung der sozialen Teilhabe des Kindes zu den verschiedenen Familienmitgliedern, der Peergroup, in der Schule/Kindergarten sowie in der Freizeit. Zu jedem Bereich wird die Frage gestellt: „Wie schätzen Sie die Teilhabe Ihres Kindes in der Schule bzw. der Freizeit ein?“ bzw. „Wie schätzen Sie die Beziehung Ihres Kindes zu Ihnen/dem anderen Elternteil/seinen Freunden/Gleichaltrigen ein?“. Die Einstufung erfolgt in 6 Stufen (0 = nicht beeinträchtigt, 1 = gering beeinträchtigt, 2 = ein wenig beeinträchtigt, 3 = teilweise beeinträchtigt, 4 = weitgehend beeinträchtigt, 5 = fast vollständig beeinträchtigt und 6 = vollständig beeinträchtigt).

#### Psychische Belastung der Eltern

Es wurde die deutsche Fassung des Brief Symptom Inventory (BSI; [3]) und eine Kurzversion der Symptomchecklist – 90R (SCL-90R; [2]) eingesetzt. Anhand einer 5‑stufigen Skala (0 = überhaupt nicht, 1 = ein wenig, 2 = ziemlich, 3 = stark, 4 = sehr stark) wird auf 9 Skalen die Symptombelastung der Befragten in den letzten 7 Tagen erfasst. Für den BSI liegen drei globale Indizes zur Beschreibung der psychischen Belastung auf aggregierter Ebene vor. Für die Analysen wurde nur der „Global Severity Index“ (GSI) verwendet. Dieser besteht aus der Summe der Itemscores dividiert durch die Anzahl der beantworteten Items und gibt die Schwere der psychischen Belastung an.

### Statistische Analyse

Für alle statistischen Analysen haben wir R 4.0.2 (Posit, Boston, Massachusetts, USA) verwendet. Zunächst erfolgte eine deskriptive Analyse der Daten. Es wurden absolute Häufigkeiten und Prozentangaben sowie Mittelwerte und Standardabweichungen berechnet. Dann wurde mit nonparametrischen Signifikanztests (χ^2^-Test nach Pearson) untersucht, ob es einen Zusammenhang zwischen dem Studienort und den in Anspruch genommenen Hilfen gibt.

Zur Ermittlung weiterer Einflussfaktoren für das Erhalten hochschwelliger Hilfen aus dem SGB VIII bzw. SGB V wurde in einem nächsten Schritt mittels binominaler logistischer Regressionsanalysen untersucht, ob neben dem Studienort, die von den Eltern wahrgenommenen psychischen Auffälligkeiten der Kinder (Gesamtproblemscore des SDQ), ihre Lebensqualität (LQO-28 des ILK) und die psychische Belastung der Eltern (GSI) relevante Prädiktoren sind. Für die Teilhabebeeinträchtigung wurde aufgrund der Ergebnisse der deskriptiven Analyse aus dem Mittelwert der Werte der Beeinträchtigung der Beziehung zur Mutter und der Beziehung zum Vater eine Eltern-Variable gebildet, die als Prädiktor in die Analyse einging. Die Voraussetzungen zur Berechnung einer Regressionsanalyse waren erfüllt. Fehlende Werte wurden nicht imputiert, sondern aus der Analyse ausgeschlossen (Studienort: 0; SDQ: 0; LQ0-28: 57, 36 %; GSI: 16, 10 %; THB Eltern: 25, 15,6 %). Für die abhängigen Variablen „hochschwellige Hilfen aus SGB VIII“, „hochschwellige Hilfen aus SGB V“ und „hochschwellige Hilfen insgesamt“ erfolgte die Modellierung jeweils schrittweise: Zunächst wurden die ausgewählten Prädiktoren univariat auf Signifikanz geprüft (Signifikanzniveau = α = 0,05, Likelihood-Ratio). Signifikante Prädiktoren wurden anschließend in ein multivariates Modell aufgenommen und somit der Effekt für konfundierende Einflüsse entsprechend adjustiert.

Mit den qualitativen Daten der Befragung nach den Wünschen wurde nach dem Verfahren der qualitativen Inhaltsanalyse ein induktives Kategoriensystem erstellt.

## Ergebnisse

### Stichprobe

In Augsburg zeigte sich, dass 63 % der infrage kommenden Eltern nicht teilnehmen wollten. Von denen, die einer Teilnahme an der Studie zunächst zugestimmt hatten, waren rund 42,7 % dann doch nicht mehr zu einem Interview bereit.

Letztendlich nahmen insgesamt 160 Patienten und Patientinnen (*n* = 100 Frauen, 62,5 %) an der Studie teil (Augsburg, städtische Region, *n* *=* 90; Neuruppin und Rüdersdorf – zusammengefasst berichtet unter „Brandenburg“, ländliche Region – *n* *=* *70*). Das Durchschnittsalter der Befragten lag bei 38 Jahren (SD = 7,03*; *Altersspanne 22–55 Jahre). In Augsburg war rund die Hälfte der Befragten weiblich, in Brandenburg waren es 80 %. Die Anzahl der Kinder zwischen 1 und 18 Jahren betrug an beiden Standorten zwischen 1 und 4 Kindern. Im Schnitt hatten die Befragten 1,8 Kinder, die im Schnitt 9 bzw. 10 Jahre alt waren. In Brandenburg war fast ein Drittel der Befragten alleinerziehend (30,0 %), in Augsburg waren es nur 11,1 %. In Brandenburg lebten bei 17,1 % der Befragten die Kinder nicht im Haushalt, in Augsburg lebte über ein Drittel der Befragten (37,8 %) ohne die Kinder im Haushalt.

Die Befragten hatten zwischen 1 und 51 Klinikaufenthalte (Augsburg M = 6; Brandenburg M = 3). In Brandenburg war knapp ein Drittel der Befragten arbeitslos (M = 27, 1 %), in Augsburg war etwas mehr als die Hälfte der Befragten arbeitslos (M = 55,6 %). An beiden Standorten lebten die Kinder mehrheitlich bei der Mutter (Augsburg: 41,1 %; Brandenburg: 51,4 %) und zu knapp 30 % mit beiden Elternteilen zusammen. 14 % der Kinder der befragten Eltern lebten in Augsburg in einer stationären Einrichtung der Jugendhilfe, in Brandenburg sind es 18 %. Bei anderen Verwandten wie den Großeltern oder der Tante lebten in Augsburg 7 % der Kinder, in Brandenburg waren es 14 %.

Etwa 40 % der EhepartnerInnen hatten nach Angabe der Befragten ebenfalls eine psychische Störung (Augsburg 41 %, Brandenburg 39 %).

Am häufigsten wurden PatientInnen befragt, die als Erstdiagnose eine Substanzmittelabhängigkeit (F1; 42,4 %) oder eine affektive Störung (F3) (41,8 %) aufwiesen (Tab. 1). Dabei waren die Befragten mit der Diagnose der Substanzmittelabhängigkeit (F1) als Erstdiagnose mit 54 % stärker in Augsburg vertreten und die Befragten mit einer affektiven Störung (F3) mit 54 % als Erstdiagnose stärker in Brandenburg.

**Table 1:** 

Diagnose		Augsburg (%)	Brandenburg (%)
F1:	Psychische Störungen und Verhaltensstörungen aufgrund des Konsums psychoaktiver Substanzen	54	25
F2:	Schizophrenie, schizotypische und wahnhafte Störungen	6	4
F3:	Affektive Störungen	33	54
F4:	Neurotische, stressbedingte und somatoforme Störungen	6	9
F5:	Verhaltenssyndrome in Verbindung mit physiologischen Störungen und physischen Faktoren	1	0
F6:	Störungen der Persönlichkeit und des Verhaltens von Erwachsenen	0	7

### Deskriptive Ergebnisse

#### Kindbezogene Faktoren

Bei der Einschätzung der psychischen Auffälligkeiten der Kinder durch die Eltern ergab sich ein mittlerer SDQ-Gesamtproblemscore von M = 12,26 (SD = 7,83, *n* = 125, Range 0–33) und 39,1 % (*n* *=* 58) der Werte, die mindestens den Cut-off für geringfügig abnormes Verhalten überschreiten (13 = < 12,8 % < 16,16 < 26,3 %). (Im Vergleich dazu die Referenzstichprobe der KIGGS-Studie: M = 8,7, SE = 0,09, 20,2 % mindestens geringfügig abnormes Verhalten [7].) Bei der Erfassung der Lebensqualität liegt der Cut-off für eine unterdurchschnittliche Lebensqualität bei Prozentrang ≤ 15, für eine überdurchschnittliche Lebensqualität bei Prozentrang ≥ 85 [19]. In der vorliegenden Stichprobe weisen die Werte auf eine recht hohe Lebensqualität hin (M = 20,84, SD = 4,84, *n* *=* 103). Dementsprechend lagen 48,5 % der Werte über dem Cut-off. Bezüglich der Beeinträchtigung in der sozialen Teilhabe gaben die befragten Eltern die größte Beeinträchtigung für die Beziehung zwischen Kindern und Vätern (M = 2,79, SD = 2,07) und Müttern (M = 1,86, SD *=* 1,73) sowie für den Bereich Schule (M = 1,75, SD = 1,77) an. Die Beziehung zu den Geschwistern (M = 1,36, SD = 1,70) und zu Gleichaltrigen (M = 1,26, SD = 1,64) wurde im Durchschnitt als gering bis ein wenig beeinträchtigt und die Teilhabe im Freizeitbereich (M = 0,93, SD = 1,35) als geringfügig beeinträchtigt eingeschätzt.

#### Elternbezogene Faktoren

Die Einschätzung der psychischen Belastung der Eltern (GSI) ergab einen Mittelwert von M = 1,49 (SD = 0,89*, n* *=* *139, *Range 0–3,79*)*. Diese Ergebnisse weisen darauf hin, dass sich die Befragten selbst als belastet erleben.

### Inferenzstatistische Ergebnisse

#### Inanspruchnahme von Hilfen

In Tab. 2 sind die Häufigkeiten der in Anspruch genommenen Hilfen dargestellt. Am meisten von den Patienten und Patientinnen in Anspruch genommen wurde soziale Unterstützung durch Familie und Freunde (*65,6* *%*) und ambulante Hilfen des SGB V (63,7 %), gefolgt von niederschwelligen und sehr niederschwelligen Hilfen des SGB VIII (46,9 und 46,3 %).

**Table 2:** 

Intensität der Hilfe	Prozentsatz der Nutzung (*n*)		
	Gesamt	Augsburg	Brandenburg
*Sozialgesetzbuch VIII (z.* *B. Jugendhilfe, Hilfen zur Erziehung)*			
Sehr niederschwellige Hilfen	46,3 (74)	50,0 (45)	41,4 (29)
Niederschwellige Hilfen	46,9 (75)	48,9 (44)	44,3 (31)
Mittelschwellige Hilfen	32,5 (52)	34,4 (31)	30,0 (21)
*Hochschwellige Hilfen*	*21,3 (34)*	27,8 (25)*	12,9 (9)*
*Sozialgesetzbuch V (z.* *B. Medizinische Behandlung)*			
Ambulante Hilfen (mittelschwellige Hilfen)	63,7 (102)	62,2 (56)	65,7 (46)
*(Teil‑)Stationäre Hilfen (hochschwellige Hilfen)*	*34,4 (55)*	41,1 (37)*	25,7 (18)*
*Andere Sozialgesetzbücher (z.* *B. VI, XII: z.* *B. Rehabilitation, Unterstützung für Behinderte)*			
*Niederschwellige Hilfen*	*33,8 (54)*	26,7 (24)*	42,9 (30)*
Mittelschwellige Hilfen	0,6 (1)	1,1 (1)	–
Hochschwellige Hilfen	5,0 (8)	6,7 (6)	2,9 (2)
*Sonstige Unterstützung (z.* *B. Familie, Freizeitaktivitäten)*			
Soziale Unterstützung	65,6 (105)	60,0 (54)	72,9 (51)
Freizeitaktivitäten	15,0 (24)	15,6 (14)	14,3 (10)

Bei der Inanspruchnahme hochschwelliger Hilfen insgesamt sind signifikante Unterschiede zwischen den Studienorten zu verzeichnen (χ^2^ *=* 8,52*, p* *=* 0,04). Der Unterschied zwischen den Studienorten ist sowohl bei der Inanspruchnahme hochschwelliger SGB-VIII-Hilfen (χ^2^ *=* 4,38*, p* *=* 0,04) als auch für hochschwellige SGB-V-Hilfen (χ^2^ *=* 4,14*, p* *=* 0,04) signifikant zuungunsten der ländlichen Region. Signifikant unterschiedlich ist auch die Inanspruchnahme niederschwelliger Hilfen anderer Sozialgesetzbücher (z. B. SGB VI, XII) allerdings zuungunsten der städtischen Region (χ^2^ *=* 8,53*, p* *=* 0,04). Bei den Berechnungen zur sozialen Teilhabe konnten positive Zusammenhänge zwischen der Inanspruchnahme von Hilfen und der Teilhabebeeinträchtigung der Kinder zu ihren Eltern gefunden werden. Das heißt, je höher die Beeinträchtigung der Beziehung der Kinder zu ihren Eltern eingeschätzt wurde, desto eher wurden hochschwellige Hilfen in Anspruch genommen. Dieser Effekt gilt sowohl für die hochschwelligen Hilfen des SGB V (χ^2^ *=* 2,12*; p* *=* 0,02*), *die hochschwelligen Hilfen des SGB VIII (χ^2^ *=* 40,76*; p* = 0,00003) und die hochschwelligen Hilfen insgesamt (χ^2^ *=* 24,05*; p* = 0,01)*.*

Die Ergebnisse der univariaten logistischen Regressionsmodelle unterstützen die Ergebnisse der χ^2^-Tests (Tab. 3). Demnach hat die Region, in der die Befragten leben, einen signifikanten Einfluss auf die Inanspruchnahme hochschwelliger Hilfen. Die Wahrscheinlichkeit, diese Hilfen in Anspruch zu nehmen, ist in Brandenburg geringer als in Augsburg (Odds Ratio SGB VIII = 0,38, Odds Ratio SGB V = 0,5). Auch ist die Wahrscheinlichkeit, hochschwellige Hilfen in Anspruch zu nehmen, für Kinder mit einem auffälligen SDQ-Gesamtproblemscore höher als für Kinder mit einem unauffälligen Problemscore. Für die Inanspruchnahme dieser Hilfen zeigt sich auch ein Zusammenhang mit der Lebensqualität der Kinder: Je geringer die eingeschätzte Lebensqualität der Kinder ausfällt, desto höher ist die Wahrscheinlichkeit, hochschwellige Hilfen in Anspruch zu nehmen.

**Table 3:** 

AV = SGB VIII	Univariat			Multivariat		
	OR	CI („lower–upper“)	*p*	OR	CI („lower–upper“)	*p*
*Studienort*	*0,38*	*0,16–0,86*	0,025*	*0,29*	*0,08–0,98*	0,05*
SDQ	1,75	0,76–3,90	0,18	–	–	–
*LQ*	*0,87*	*0,78–0,97*	0,012*	0,89	0,78–1,01	0,07
GSI	1,13	0,50–2,57	0,771	–	–	–
*Teilhabe Eltern*	*2,32*	*1,67–3,41*	0,000003*	1,35	2,17	0,21
AV = SGB V	Univariat			Multivariat		
	OR	CI („lower–upper“)	*p*	OR	CI („lower–upper“)	*p*
*Studienort*	*0,5*	*0,25–1,0*	0,04*	0,45	0,17–1,12	0,09
*SDQ*	*2,16*	*1,04–4,46*	0,04*	2,83	0,96–8,55	0,06
*LQ*	*0,90*	*0,82–1,0*	0,02*	0,96	0,85–1,08	0,48
GSI	0,87	0,43–1,73	0,68	–	–	–
*Teilhabe Eltern*	*1,39*	*1,10–1,78*	0,01*	1,24	1,78	0,25
AV = hochschwellige Hilfen insgesamt	Univariat			Multivariat		
	OR	CI („lower–upper“)	p	OR	CI („lower–upper“)	*p*
*Studienort*	*0,49*	*0,25–0,92*	0,03*	0,51	0,2–1,25	0,15
*SDQ*	*2,46*	*1,20–5,15*	0,02*	*3,09*	*1,08–9,09*	0,03*
*LQ*	*0,89*	*0,81–0,92*	0,01*	0,95	0,85–1,07	0,42
GSI	0,89	0,46–1,72	0,73	–	–	–
*Teilhabe Eltern*	*1,71*	*1,34–2,25*	0,00*	1,16	1,64	0,41

In der multivariaten logistischen Regressionsanalyse zeigt sich, dass nach Adjustierung für Confounding bei der Inanspruchnahme hochschwelliger Hilfen des SGB VIII der Effekt des Studienorts weiterhin signifikant ist. Bei der Inanspruchnahme hochschwelliger Hilfen insgesamt bleibt der Effekt der psychischen Auffälligkeit der Kinder signifikant.

### Qualitative Ergebnisse

Bei den erfassten Wünschen nach Hilfsangeboten (*n* = 153) zeigt sich, dass die Mehrzahl der Wünsche sich auf Unterstützungsangebote aus dem SGB VIII bezieht (39,9 %,* n* *=* 61). Insgesamt wünschen sich 12,4 % (*n* = 19) der Befragten Unterstützung durch das Jugendamt, in Brandenburg sind es sogar 22,9 % (*n* = 11).

Fast ein Drittel der angegebenen Wünsche beziehen sich auf Unterstützung aus dem SGB V (31,4 %, *n* = 48). Dabei ist der Wunsch nach psychologischer Betreuung der meist genannte (Augsburg [Aug.] 6,7 %, *n* = 7; Brandenburg [BB] 22,9 %, *n* = 11). Die Aussagen dieser Kategorie sind vielfältig und adressieren sowohl die Seelsorge im Krankenhaus, psychologische Betreuung speziell bei Wochenbettdepression als auch besser verfügbare Behandlungsmöglichkeiten sowie weitere Wünsche (Tab. 3). Am häufigsten genannt wird von den befragten psychisch erkrankten Eltern in dieser Kategorie jedoch der Wunsch nach psychologischer Betreuung für das Kind (10 Nennungen). Die Befragten in Augsburg geben zudem vermehrt an, sich Veränderungen an den Strukturen in der Klinik bez. ihres Aufenthalts zu wünschen (Einbindung der Kinder, flexiblere Regelungen, Räumlichkeiten u. W.; Tab. 4; Aug. 11,5 %, *n* = 12; BB 2,1 %,* n* = 1). Auch die bessere Vernetzung verschiedener Disziplinen ist ein mehrfach genannter Wunsch bei den Befragten in Augsburg (6,7 %,* n* = 7). Ein weiterer Wunsch ist eine bessere Informationspraxis (z. B. über bestimmte Erkrankungen, aber auch über Hilfsangebote; Aug. 3,9 %,* n* = 4; BB 2,1 %,* n* = 3).

**Table 4:** 

Kategorie	Inhalte
Abbau innerer Hemmungen	Eltern-Kind-Kur Eltern- und Familienberatung Krankenhaus Jugendamt (2-mal genannt) Psychologe
Angebote für Kinder	Grünflächen/Spielplätze
Ansprechpartner	Zur Kinderbetreuung im Notfall und Finanzen Zum Umgang (für Väter) Für (erwachsene) Kinder suchtkranker Eltern Für alleinerziehende mit kleinem Kind Bei „Ämtergängen“ Bei finanziellen Angelegenheiten
Beratung	Zu Paarkonflikten (3-mal genannt) Zum Umgang Zur Erziehung Für Kinder psychisch kranker Eltern
Bessere Vernetzung/Zusammenarbeit	Kommunikation zwischen den Stationen Einbindung von Hebammen bei stationärer Behandlung Vernetzung stationäre Behandlung zum niedergelassenen Gynäkologen Weiterbetreuung beim Behandelnden nach Entlassung Mehr Durchlässigkeit zwischen ambulanter und teilstationärer Behandlung Unterstützungsnetzwerke, die im Notfall aktiviert werden können Mehr Werbung für Hilfsangebote verschiedener Stellen
Finanzielle Unterstützung	Geld für „Familienerholung“ Aufstockung der Halbwaisenrente Mehr Unterstützung für Alleinerziehende Geld für Familienausflüge (3-mal genannt) Finanzielle Unterstützung bei Jobverlust Mehr Geld für Kleidung und Spielsachen Mehr Geld für Schulausrüstung Beratung wegen Umzugskostenhilfe Mehr finanzielle Unterstützung (2-mal genannt)
Freizeitgestaltung	Bessere Information zu Ferienprogrammen Programm sollte besser bezahlbar sein Günstigere und flächendeckendere Angebote (2-mal genannt) Zu wenig Angebote für Jugendliche Kurse an Kindergarten anschließen Im Jugendzentrum auch für jüngere Kinder Mehr Angebote bei offenen Ganztagsklassen (2-mal genannt) Mehr Jugendzentren Sport (2-mal genannt) Musikschule und Kinderfeuerwehr
Haushaltshilfe	Haushaltshilfe (4-mal genannt)
Information	Zu Erkrankungen (2-mal genannt) Im Behandlerteam teilweise uneinheitlich Zur Mutterschaft Zu Hilfsangeboten (2-mal genannt)
Kinderbetreuung	Babysitter Hort Personal Kosten Notfall Öffnungszeiten Kita
Kommunikation in der Familie	Erziehung Umgang (2-mal genannt) Aussöhnung
Kontrolle durch Jugendamt	Bei auffälligen Familien (3-mal genannt)
Patenprogramme	Leihoma Patenprogramme (2-mal genannt)
Psychologische Betreuung	Seelsorge im Krankenhaus Wochenbettdepression Selbsthilfegruppe Mehr Unterstützung durch Schulpsychologie Besser verfügbare Behandlungsmöglichkeiten (2-mal genannt) Für das Kind (10-mal genannt) Anständige Therapeuten Angehörigensorge
Schulische Unterstützung	Kosten Schulbegleitung
Strukturen Klinikaufenthalt	Räumlichkeiten (4-mal genannt) Flexiblere Regelungen Rahmenbedingungen stationäre Therapie (5-mal genannt) Bessere Einbindung der Kinder (3-mal genannt)
Symptomverbesserung	Bessere Stimmung, stabil/trocken werden (6-mal genannt) Methadon Legalisieren von Opiaten und THC
Unterstützung	Unterstützung bei der Jobvermittlung Unterstützung bei der Wohnungssuche
Unterstützung durch die Familie	Durch Vater/Ehemann (4-mal genannt) Durch die Großeltern (4-mal genannt) Durch Familie/soziales Umfeld allgemein (3-mal genannt)
Unterstützung durch das Jugendamt	Familienhilfe Begleitete Umgänge (2-mal genannt) Ambulante Hilfe Standardbetreuung für alle Trennungsfamilien Bei der Vermittlung von Hilfsangeboten Generell mehr Hilfe/Kontakt (3-mal genannt) Hilfe bei Beziehung zum Kind Möglichkeit, den zuständigen Mitarbeiter zu wechseln Hilfe wurde abgewiesen Einzelfallhilfe (7-mal genannt)
Wohnungslosenhilfe	Angebote speziell für Frauen
Zusammenarbeit mit dem Jugendamt auf Augenhöhe	Vor Inobhutnahme genauer prüfen, ob notwendig Mehr mit den Eltern abstimmen Wünsche der Jugendlichen mehr respektieren

Bei den sonstigen Sozialgesetzbüchern wird der Wunsch nach finanzieller Unterstützung angegeben, vor allem in Augsburg (10,6 %* n* = 11; BB 4,2 %,* n* = 2). An beiden Studienorten, besonders aber in Brandenburg, wird der Wunsch nach Unterstützung durch die Familie mehrfach genannt (Aug. 4,8 %,* n* = 5; BB 10,4 %,* n* = 5) ebenso wie der Abbau von inneren Hemmschwellen und Vorurteilen (insgesamt 3,3 %,* n* = 5). Dies bezieht sich bereichsübergreifend sowohl auf das Jugendamt, Elternberatung, die Klinik und Psychologen. Eine bessere Zusammenarbeit wird von den Eltern vor allem im Gesundheitswesen und ausschließlich in Augsburg gewünscht (insgesamt 4,6 %,* n* = 7).

## Diskussion

Die Studie untersuchte die Inanspruchnahme von Hilfen und mögliche Einflussfaktoren durch psychisch kranke Eltern für ihre Kinder in klinischen Populationen in zwei verschiedenen Regionen in Deutschland. Es handelte sich um schwer erkrankte Eltern, die (teil)stationäre Krankenhausbehandlung in Anspruch nahmen, was sich auch in den verwendeten Instrumenten zeigte. Auch die Kinder der befragten Eltern waren nach deren Urteil im SDQ stärker belastetet als Kinder der Referenzstichprobe [7, 16, 24].

Hinsichtlich der Hauptfragestellung, ob und welche Hilfen in Anspruch genommen werden, zeigte sich, dass eine hohe Zahl der psychisch (schwer) erkrankten Eltern Hilfe und Unterstützung erhalten. Allerdings stellt weiterhin das nahe Umfeld der Erkrankten (Freunde und Familie) offenbar die hauptsächliche Unterstützung dar. Jedoch nutzt auch ein erheblicher Teil der Befragten Hilfen aus dem Gesundheitssystem. Die Ergebnisse zeigen, dass schwerer beeinträchtigte Kinder mehr hochschwellige Hilfen erhalten und dass insbesondere bei hochschwelligen Hilfen aus dem Bereich des SGB VIII der Wohnort einen signifikanten Einfluss hat. Andere Studien haben hierzu andere Ergebnisse gefunden: Schunke et al. fanden keinen Zusammenhang zwischen den Verhaltensauffälligkeiten der Kinder und der Inanspruchnahme von Hilfen [27]. Unsere Ergebnisse weisen darauf hin, dass Eltern, die ihre Kinder als auffälliger wahrnehmen, auch mehr Hilfen in Anspruch nehmen. Nach Wahl et al. sind relevante Einflussfaktoren für die Inanspruchnahme von Hilfen deren Erreichbarkeit und Verfügbarkeit [29]. Hier zeigte unsere Studie, dass durchaus regionale Unterschiede in der Inanspruchnahme bestehen.

Auch wenn Befunde der Literatur zeigen, dass Wissen besonders über spezifische Hilfsangebote für psychisch kranke Eltern fehlt bzw. Angebote nicht angenommen werden, weil sie nicht bekannt sind (vgl.; [6, 13, 29]), spiegelt sich dies nicht in den Wünschen der Befragten dieser Stichprobe wider. Nur 2 von 153 Nennungen beziehen sich auf die Information über Hilfsangebote (1,31 %). Mehr Bedarf gibt es bei der Bearbeitung innerer Hemmungen, Ängste und Vorurteile (3,3 %). Den Befunden anderer Studien entsprechend, scheinen diese der Inanspruchnahme einiger Hilfen entgegenzustehen [6]. Dabei beziehen sich diese Widerstände nicht nur auf „Ressentiments gegen das Jugendamt“ [11], sondern disziplinübergreifend auf den Klinikaufenthalt, die Erziehungs- und Familienberatung, Psychologinnen und die Inanspruchnahme einer Eltern-Kind-Kur. Der Wunsch nach mehr Unterstützung durch das Jugendamt ist – besonders in Brandenburg – groß (22,9 %). Dieser Befund könnte daraufhin deuten, dass die bisherige Unterstützung durch die Jugendämter in einem Flächenland wie Brandenburg die betroffenen Familien weniger gut erreicht. Eine andere mögliche Erklärung für diesen Befund könnte in der Verteilung der Stichprobe liegen, da diese in Brandenburg mehr Mütter beinhaltete, mehr Personen mit im Haushalt lebenden Kindern, mehr alleinerziehende und mehr Personen mit affektiven Störungen. Möglicherweise wünscht sich gerade diese Gruppe mehr Unterstützung durch das Jugendamt. Eine bessere Zusammenarbeit und Vernetzung wird insbesondere im Gesundheitswesen gewünscht, was den Ergebnissen von Hefti et al. entspricht [6]. Eine bessere Vernetzung der Disziplinen und Zusammenarbeit im Gesundheitswesen, insbesondere zwischen den „Psych-Fächern“ und der Somatik, empfiehlt auch die Regierungskommission für eine moderne und bedarfsgerechte Krankenhausversorgung in ihrer achten Stellungnahme [23]. Auch die regionalen Unterschiede in der Versorgungslandschaft der Bundesrepublik allgemein werden hier adressiert. Der Ausbau der kinder- und jugendpsychiatrischen Versorgung, insbesondere in den unterversorgten Gebieten, auch im ambulanten Bereich, wird in der Stellungnahme ebenso gefordert wie eine bessere Verzahnung zwischen der Kinder- und Jugendpsychiatrie und der Erwachsenenpsychiatrie [ebd.].

Leichte Zugänge zu Hilfen und Familien frühzeitig, niederschwellig und direkt zu erreichen fordert auch die Arbeitsgruppe psychisch und suchtkranker Eltern in ihrem Abschlussbericht vom Februar 2020 [1]. Zudem soll es eine Lotsenfunktion geben, die das Zusammenwirken professioneller Akteure aus unterschiedlichen Unterstützungssystemen koordiniert und einen Überblick über die vorhandenen Leistungen und Angebote hat [ebd.]. Denn Angebote, auch spezifisch für Kinder psychisch kranker Eltern, gibt es in der Bundesrepublik. Sie sind aber meist nicht in der Lebenswelt der Betroffenen angesiedelt, weshalb sich auch hier die Frage nach den Zugangswegen stellt [4].

Weitere Forschung sollte systemübergreifend Hilfen und deren Inanspruchnahme in den Blick nehmen und schauen, inwieweit die Wünsche und Veränderungsbedarfe der betroffenen Familien realisierbar oder eventuell auch schon realisiert, aber nicht bekannt sind. Hierbei sollte besonders darauf geachtet werden, ob die Schwerpunkte der jeweils anderen Disziplinen bekannt sind (also beispielsweise die Erkrankung des Elternteils bei SGB-VIII-Hilfen für das Kind oder eine mögliche psychische Belastung oder gar Verhaltensauffälligkeit des Kindes bei SGB-V-Hilfen für das erkrankte Elternteil; [14]). Mithilfe weiterer Forschung sollten auch systematisch die durch die Krankenhausreform angestoßenen Veränderungen überprüft werden, um zu schauen, ob die benannten Verbesserungen in den Versorgungsstrukturen auch in der Realität gewinnbringend umgesetzt werden konnten.

## Limitationen der Studie

Da die Teilnahme an der Studie freiwillig war, wurden nicht alle Eltern befragt. Die meisten angefragten Eltern, die abgelehnt haben (31 %), gaben keine Gründe an, warum sie nicht an der Studie teilnehmen wollten. Genannte Gründe waren „kurz vor Entlassung“ (27 %), „Ablehnung wegen Erkrankung“ (15 %), „Betreuung notwendig“ (15 %), sprachliche Hürden (3 %). 9 % der Befragten erklärten sich zwar zum Interview bereit, verschoben den Termin dann jedoch mehrfach, sodass er schließlich nicht zustande kam. Es sind auch nicht alle Diagnosegruppen in der Stichprobe vertreten. Patienten und Patientinnen, die sich in der Akutphase der Erkrankung befanden und krankheitsbedingt nicht in der Lage waren, an dem Interview teilzunehmen, wurden nicht eingeschlossen. Eine Repräsentativität ist demnach in dieser vorliegenden recht aufwendigen und umfassenden Erhebung nicht vollständig gegeben. Die Werte zum Verhalten, der Lebensqualität und der Teilhabebeeinträchtigung der Kinder wurden ausschließlich in der Fremdbeurteilung erfasst, eine Diagnostik der Kinder fand im Rahmen der Studie nicht statt. Die Inanspruchnahme von Hilfen wurde allein anhand des Selbstberichts der Patienten und Patientinnen erfasst, ob Hilfen vergessen oder aus anderen Gründen nicht benannt wurden, kann nicht festgestellt werden. Auch können Faktoren der sozialen Erwünschtheit, die die Ergebnisse möglicherweise positiv verzerren, nicht ausgeschlossen werden.

## Schlussbetrachtung

Die Ergebnisse legen nahe, dass in den beiden Studienregionen in Augsburg und Brandenburg Familien mit einem psychisch kranken Elternteil Hilfen erhalten: Diese insgesamt positiven Ergebnisse könnten auch Folge davon sein, dass die Thematik psychisch erkrankter Eltern verstärkt in die öffentliche Wahrnehmung sowie in die Wahrnehmung in den Hilfesystemen gerückt ist. Dennoch zeigen sich regionale Unterschiede, insbesondere in der Inanspruchnahme der hochschwelligen Hilfen aus dem SGB VIII.

## Fazit für die Praxis

- Familien mit einer psychischen Erkrankung erhalten Hilfen aus dem deutschen Versorgungssystem.
- Die am meisten benannten Hilfen sind Hilfen durch die Familie und Freunde sowie Hilfsangebote aus dem SGB V.
- Familien mit als stärker psychisch auffällig eingeschätzten Kindern oder, bei denen die Beziehung zu den Eltern stärker eingeschränkt ist, erhalten häufiger hochschwellige Hilfen, was dafür spricht, dass Hilfebedarfe in den unterschiedlichen Systemen erkannt werden.
- Es gibt regionale Unterschiede bei der Art der in Anspruch genommenen Hilfen.
- Entgegen früherer Ergebnisse scheint die Sorge gegenüber dem Jugendamt geringer zu sein, da viele Befragte sich Unterstützungsangebote aus dem SGB VIII wie Beratungsangebote und insbesondere mehr Unterstützung durch das Jugendamt wünschten.
- Es gibt regionale Unterschiede bei der Benennung der Wünsche nach bestimmten Unterstützungsangeboten.
- Zukünftige Erhebungen sollten hinsichtlich der vielfältigen Hilfen, die psychisch erkrankte Eltern erhalten, untersuchen, inwieweit diese Hilfen koordiniert sind und ob z. B. auch bei Hilfen außerhalb des SGB V die psychische Erkrankung des Elternteils bekannt ist.

## Footnotes

1 Zwönitzer A, Ziegenhain U, Künster A (2012) Unveröffentlichter Interviewleitfaden zur Erhebung erhaltener Unterstützungsangebote.
