# Erratum zu: Unterstützung für Familien mit einem psychisch erkrankten Elternteil: Welche Hilfen nehmen psychisch erkrankte Eltern in Anspruch und welche wünschen sie sich?

**DOI:** 10.1007/s00115-024-01617-5

**Published:** 2024-02-26

**Authors:** Stefanie Bienioschek, Daria Nolkemper, Jennifer Schroth, Joachim Behr, Martin Heinze, Ute Ziegenhain, Max Schmauss, Michael Kölch

**Affiliations:** 1 Klinik für Psychiatrie, Psychotherapie und Psychosomatik im Kindes- und Jugendalter, Universitätsklinikum Ruppin-Brandenburg, Fehrbelliner Str. 38, 16816 Neuruppin, Deutschland; 2 Medizinische Hochschule Brandenburg, Neuruppin, Deutschland; 3 Klinik für Kinder- und Jugendpsychiatrie und Psychotherapie, Universitätsklinikum Ulm, Ulm, Deutschland; 4 Klinik für Psychiatrie, Psychotherapie und Psychosomatik, Universitätsklinikum Ruppin-Brandenburg, Medizinische Hochschule Brandenburg Theodor Fontane, Neuruppin, Deutschland; 5 Klinik für Psychiatrie und Psychotherapie, Campus Mitte, Charité – Universitätsmedizin Berlin, Berlin, Deutschland; 6 Fakultät für Gesundheitswissenschaften, Gemeinsame Fakultät der Universität Potsdam, der Medizinischen Hochschule Brandenburg Theodor Fontane und der Brandenburgischen Technischen Universität Cottbus-Senftenberg, Potsdam, Neuruppin, Cottbus, Deutschland; 7 Zentrum für seelische Gesundheit, Immanuel Klinik Rüdersdorf, Universitätsklinikum der MHB, Rüdersdorf, Deutschland; 8 Klinik für Psychiatrie, Psychotherapie und Psychosomatik, Universität Augsburg, Augsburg, Deutschland; 9 Klinik für Psychiatrie, Neurologie, Psychosomatik und Psychotherapie im Kindes- und Jugendalter, Universitätsmedizin Rostock, Rostock, Deutschland

**Erratum zu:**

**Nervenarzt 2023**

10.1007/s00115-023-01584-3

Die ursprünglich veröffentlichte Version enthielt Fehler in der Korrespondenzanschrift und in Affiliation ^7^. Es wurde wie folgt angegeben:

^7^ Psychiatrie, Psychotherapie und Psychosomatik, Immanuel Klinik Rüdersdorf, Universitätsklinikum der MHB, Rüdersdorf, Deutschland

Korrespondenzadresse

Prof. Dr. med. Stefanie Bienioschek

Klinik für Psychiatrie, Psychotherapie und Psychosomatik im Kindes- und Jugendalter, Universitätsklinikum Ruppin-Brandenburg

Fehrbelliner Str. 38, 16816 Neuruppin,

Deutschland

stefanie.bienioschek@mhb-fontane.de

Diese wurden korrigiert und lauten nun wie folgt:

^7^ Zentrum für seelische Gesundheit, Immanuel Klinik Rüdersdorf, Universitätsklinikum der MHB, Rüdersdorf, Deutschland

Korrespondenzadresse

Dr. med. Stefanie Bienioschek

Klinik für Psychiatrie, Psychotherapie und Psychosomatik im Kindes- und Jugendalter, Universitätsklinikum Ruppin-Brandenburg

Fehrbelliner Str. 38, 16816 Neuruppin,

Deutschland

stefanie.bienioschek@mhb-fontane.de

Der Originalbeitrag wurde korrigiert.

